# PGC-1β: A Regulator of Mitochondrial Function with Subtle Roles in Energy Metabolism

**DOI:** 10.1371/journal.pbio.0040402

**Published:** 2006-11-07

**Authors:** Françoise Chanut

The evolutionary success of mammals is owed in part to their ability to adjust their metabolism to the demands of their environment. For instance, mice faced with dropping external temperatures crank up the output of a specialized type of fat—called brown adipose tissue (BAT) —that generates heat. Faced with a lack of food, they turn on the production of glucose, a highly energetic sugar, by the liver. Both of these adaptive responses depend on a nuclear protein called PGC-1α. PGC-1α simultaneously increases the expression of genes acting at multiple steps along the heat- or glucose-production pathways, thereby quickly boosting the pathways’ efficacy. A related protein, PGC-1β, shares many of PGC-1α’s functional characteristics: for instance, both proteins can, when overexpressed in mice, increase the activity of mitochondria, the intracellular organelles that turn sugars and fats into heat or the cellular fuel ATP. To determine what role PGC-1β normally plays in energy metabolism, Christopher Lelliott, Gema Medina-Gomez, Antonio Vidal-Puig, and their colleagues created mice lacking the PGC-1β gene. Their recent study of the resulting phenotype shows that PGC-1β has different functions than PGC-1α and plays a subtler role than PGC-1α in energy metabolism.

As expected from PGC-1β’s ability to stimulate mitochondrial function, the loss of PGC-1β decreased the transcription of genes encoding many of the mitochondrial proteins that generate ATP and heat. Yet the mutant mice appeared essentially healthy under normal conditions. Still, a slight impairment of mitochondrial function might lead to some metabolic imbalance, for instance obesity, since mitochondria burn energy that would otherwise be stored as fat. But the mutant mice were in fact smaller and leaner than their wild-type counterparts. This phenotype did not reflect differences in appetite or digestion, since the mutants ate and eliminated similar amounts of food as wild types. Instead, the mutants had higher metabolic rates than wild types at ambient temperature: they burnt more calories than wild types did to sustain basal physiological functions such as respiration, heartbeat, and body temperature, which would suggest that their mitochondrial functions were increased, rather than decreased.

The explanation for these counterintuitive findings is that loss of PGC-1β led to increased expression of PGC-1α in fat tissues. The researchers propose that the increase in PGC-1α, which can stimulate heat production in BAT, compensates for the loss of PGC-1β at ambient temperature. To remove the compensatory effect of PGC-1α, the researchers measured the metabolic rate of mutants housed at 30 °C, a temperature close to body temperature, at which PGC-1α expression is largely abolished. At this temperature, mice lacking PGC-1β had a reduced metabolic rate, suggesting that PGC-1β can contribute to maintaining normal metabolic rates.

**Figure:**
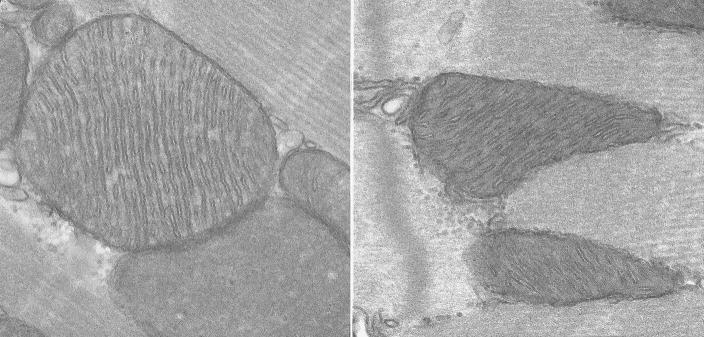
The typical mitochondrial density within hearts of normal mice (left panel) becomes considerably reduced in mice lacking the gene for PGC-1β (right panel).

The mutant mice were able to withstand and adapt to cold, presumably because cold temperatures could still trigger PGC-1α expression. However, when mutant mice that were acclimatized to 4 °C were further stimulated by norepinephrine, a neurotransmitter that normally induces BAT to respond to cold, their BAT heat output was blunted compared to that of wild-type BAT. Therefore, PGC-1β must normally play a role in thermal regulation, though its role is obscured by PGC-1α’s induction at low temperatures.

In other tissues such as liver, muscle, and heart, loss of PGC-1β did not trigger PGC-1α expression, which made the role of PGC-1β easier to discern. In the liver, PGC-1β proved necessary for the proper disposal of cholesterol and other lipid forms when mice were fed a diet rich in saturated fat. In muscles and heart, PGC-1β was found necessary to maintain a normal number of mitochondria. But even with a reduced mitochondrial count, the hearts of mice lacking PGC-1β functioned normally. Their only anomaly was a failure to increase their pulse rate properly when challenged with the heart stimulant dobutamine. By comparison, mice lacking PGC-1α develop severe heart failure.

These observations show that although PGC-1β and PGC-1α share sequence and functional similarities and are expressed in the same tissues, they cannot completely substitute for one another. The researchers propose that PGC-1β maintains basal metabolic functions, whereas PGC-1α allows the body to step up it response to high energetic demands.

